# Triple-gene deletion for osteocalcin significantly impairs the alignment of hydroxyapatite crystals and collagen in mice

**DOI:** 10.3389/fphys.2023.1136561

**Published:** 2023-03-28

**Authors:** Zihan Xu, Chao Yang, Feng Wu, Xiaowen Tan, Yaxiu Guo, Hongyu Zhang, Hailong Wang, Xiukun Sui, Zi Xu, Minbo Zhao, Siyu Jiang, Zhongquan Dai, Yinghui Li

**Affiliations:** ^1^ School of Life Science and Technology, Harbin Institute of Technology, Harbin, China; ^2^ State Key Laboratory of Space Medicine Fundamentals and Application, China Astronaut Research and Training Center, Beijing, China; ^3^ Department of Pathology and Forensics, Dalian Medical University, Dalian, China

**Keywords:** osteocalcin, collagen fiber arrangement, bone mineral composition, bone microstructure, tail suspension

## Abstract

Osteocalcin (Ocn), also known as bone Gla protein, is synthesized by osteoblasts and thought to regulate energy metabolism, testosterone synthesis and brain development. However, its function in bone is not fully understood. Mice have three Ocn genes: Bglap, Bglap2 and Bglap3. Due to the long span of these genes in the mouse genome and the low expression of Bglap3 in bone, researchers commonly use Bglap and Bglap2 knockout mice to investigate the function of Ocn. However, it is unclear whether Bglap3 has any compensatory mechanisms when Bglap and Bglap2 are knocked out. Considering the controversy surrounding the role of Ocn in bone, we constructed an Ocn-deficient mouse model by knocking out all three genes (Ocn^−/−^) and analyzed bone quality by Raman spectroscopy (RS), Scanning electron microscopy (SEM), Fourier transform infrared spectroscopy (FTIR) and MicroCT (μCT). The RS test showed that the alignment of hydroxyapatite crystals and collagen fibers was significantly poorer in Ocn^−/−^ mice than in wild-type (WT) mice. Ocn deficiency resulted in a looser surface structure of bone particles and a larger gap area proportion. FTIR analysis showed few differences in bone mineral index between WT and Ocn^−/−^ mice, while μCT analysis showed no significant difference in cortical and trabecular regions. However, under tail-suspension simulating bone loss condition, the disorder of hydroxyapatite and collagen fiber alignment in Ocn^−/−^ mice led to more obvious changes in bone mineral composition. Collectively, our results revealed that Ocn is necessary for regulating the alignment of minerals parallel to collagen fibrils.

## 1 Introduction

Bone is a biomineralized composite composed of collagen, non-collagenous proteins (NCPs) and carbonate-substituted hydroxyapatite minerals (López Barreiro et al., 2022). Among the bone matrix, osteocalcin (Ocn) is one of the most abundant NCPs. Other major types of NCPs are osteonectin (ON), fibronectin (FN), and osteopontin (OPN) (Hauschka et al., 1989). Unlike these proteins, osteocalcin is a bone-specific protein mainly produced by osteoblasts during bone formation and is found deep in the densely mineralized region of cortical bone (Weinreb et al., 1990; Chenu et al., 1994).

In humans and rats, Ocn is encoded by a single gene (*BGLAP*) (Li et al., 1995). While in mice, there are three genes: Bglap, Bglap2, which are highly expressed in bone; and Bglap3, which is mainly expressed in the kidney (Desbois et al., 1994). Post-translational γ-carboxylation carried out by γ-glutamyl carboxylase (GGCX) is one of the critical features of Ocn. During carboxylation, a second carboxyl group is added to specific glutamyl residues (Glu) at positions 17, 21, and 24 forming γ-carboxyglutamyl residues (Gla). This modification leads to a conformational change, stabilizing the α-helical portion of the protein and conferring a greater affinity for calcium and hydroxyapatite (Lee et al., 2000). Because of these structural features, Ocn is thought to play an essential role in bone mineralization processes and hydroxyapatite growth (Hoang et al., 2003). Meanwhile, Ocn is also often used as an indicator of bone turnover, which has been justified by the necessity of preserving the mechanical integrity of the skeleton and regulating calcium and phosphorus homeostasis (Lian and Gundberg, 1988; McGuigan et al., 2010).

Based on the Ocn deficient mice generated by simultaneously deleting both Bglap and Bglap2 genes, Ducy P., found that a lack of Ocn leads to an increase in bone formation without impairing bone resorption. However, this knockout of Ocn did not affect bone mineralization. Furthermore, female Ocn-deficient mice were resistant to oophorectomy-induced bone loss (Ducy et al., 1996). Total loss of Ocn in rats resulted in bones with significantly increased trabecular thickness, density, and volume. Cortical bone volume and density were not increased in null animals (Lambert et al., 2016). These results were inconsistent with the theoretically predicted functions of Ocn and brought certain difficulties to identify the role of Ocn in bone. In the follow-up studies, the Karsenty team demonstrated that uncarboxylated osteocalcin (ucOcn) had effects on energy metabolism, male fertility, brain function as well as muscle function and proposed that ucOcn is a hormone secreted by bone (Lee et al., 2007; Oury et al., 2011; Oury et al., 2013; Mera et al., 2016).

On the contrary, two recent studies reported inconsistent conclusions. Moriishi et al. (2020) constructed a new Ocn^−/−^ mouse model which was backcrossed to C57BL/6N more than 8 times before analysis, while the previous Ocn^−/−^ mice published in 1996 were analyzed in the mixed background of C57BL/6J and 129/SV. The results suggested that ocn is necessary for aligning apatite crystallites but is not involved in regulating bone quantity by analysis of mice with deleted Bglap and Bglap2 genes. Glucose metabolism, testosterone synthesis, and muscle mass are also normal in Ocn-deficient mice. Diegel CR finds alterations in the maturity of collagen and carbonate-to-phosphate ratio in cortical bone. Still, bone mass, serum glucose levels and male fertility were no different in the mice with a new Bglap and Bglap2 double-knockout (dko) allele compared with wild-type littermates (Diegel et al., 2020). The above results indicate that the role played by Ocn in the skeleton has not yet been fully elucidated. In addition, most of the existing research mainly used Bglap and Bglap2 knockout mice, but the functional significance of the Bglap3 allele in mice is also unclear and the potential contributor of the Bglap3 gene can not be ruled out.

With the development of technology, more methods could be used to assess bone microarchitecture and mineral composition. Conventional X-ray diffraction (XRD) can give direct information about the crystalline quality and size as well as the presence of defects in the crystal (Lai et al., 2016). In terms of evaluating bone mineralization, quantitative backscattered electron imaging (qBEI), quantitative magnetic resonance (QMR), micro-computed tomography (μCT), Fourier-Transform Infrared Spectroscopy (FTIR), and Raman spectroscopy analysis (RS) can be used to quantify the degree of bone mineralization and obtain bone microstructure data with different resolutions (Donnelly, 2011). Among these methods, FTIR and RS technology could provide the amount of minerals in each organic matrix unit by calculating the mineral-to-matrix ratio (MMR). The μCT process can determine the mineral density (mgHA/cm^3^) and bone microstructure (Paschalis et al., 2017). The ν_1_PO_4_ and AmideI peaks of the RS test are sensitive to the detection of the incident angle, which can provide information on the alignment direction of hydroxyapatite crystals and collagen from a microscopic perspective. In addition, RS can scan different points separately to obtain the angular relationship between hydroxyapatite and collagen at various sites. These technological advances allow researchers worldwide to analyze Ocn biology in bone at unprecedented detail.

Using μCT, Bailey et al. (2017) found that Ocn and OPN work together to maintain the quality of bone materials, and Ocn knockout has a more significant impact than OPN. A computational study was presented to elucidate the critical roles of Ocn and OPN at the nanoscale interfibrillar interface and reveals the extremely high interfacial toughness of the Ocn/OPN (Wang et al., 2020). With the help of XRD and TEM, Iline-Vul et al. (2019) suggested that the post-translationally added γ-carboxylates Ocn has a marked regulatory effect on the mineral phases coating the apatite crystallites formed. The c-axis orientation of crystallography in Ocn knockout mice was poor consistency (tested by XRD technology), which means that Ocn deficiency may affect bone microstructure (Komori, 2020; Moriishi et al., 2020).

To elucidate the function of Ocn in bone more comprehensively, we generated Ocn^−/−^ mice with simultaneous knockout of all three Ocn genes including Bglap, Bglap2, and Bglap3. Taking advantage of the property that the RS method is sensitive to the angle of hydroxyapatite and collagen we examined the correlation between hydroxyapatite and collagen micro alignment angle in the mouse femoral cortical bone area and acquired direct evidence of the effect of Ocn deficiency on bone mineral alignment. We applied FTIR and μCT to observe the effect of Ocn knockout on bone mass and quality, and Scanning electron microscopy (SEM) was used for imaging bone structure from the microscale to sub-cellular scales. Meanwhile, a tail-suspended mouse model was utilized to study the influence of Ocn on the stability of mineral components in the hindlimb unloading state. Our data showed that Ocn regulates the alignment of minerals parallel to collagen fibrils. This structural change further affects the ability of bone to maintain mineral stability, demonstrating that Ocn plays an essential role in bone structure maintenance.

## 2 Materials and methods

### 2.1 Animal experiments generation of Ocn^−/−^ mice by homologous recombination method

**FIGURE 1:**
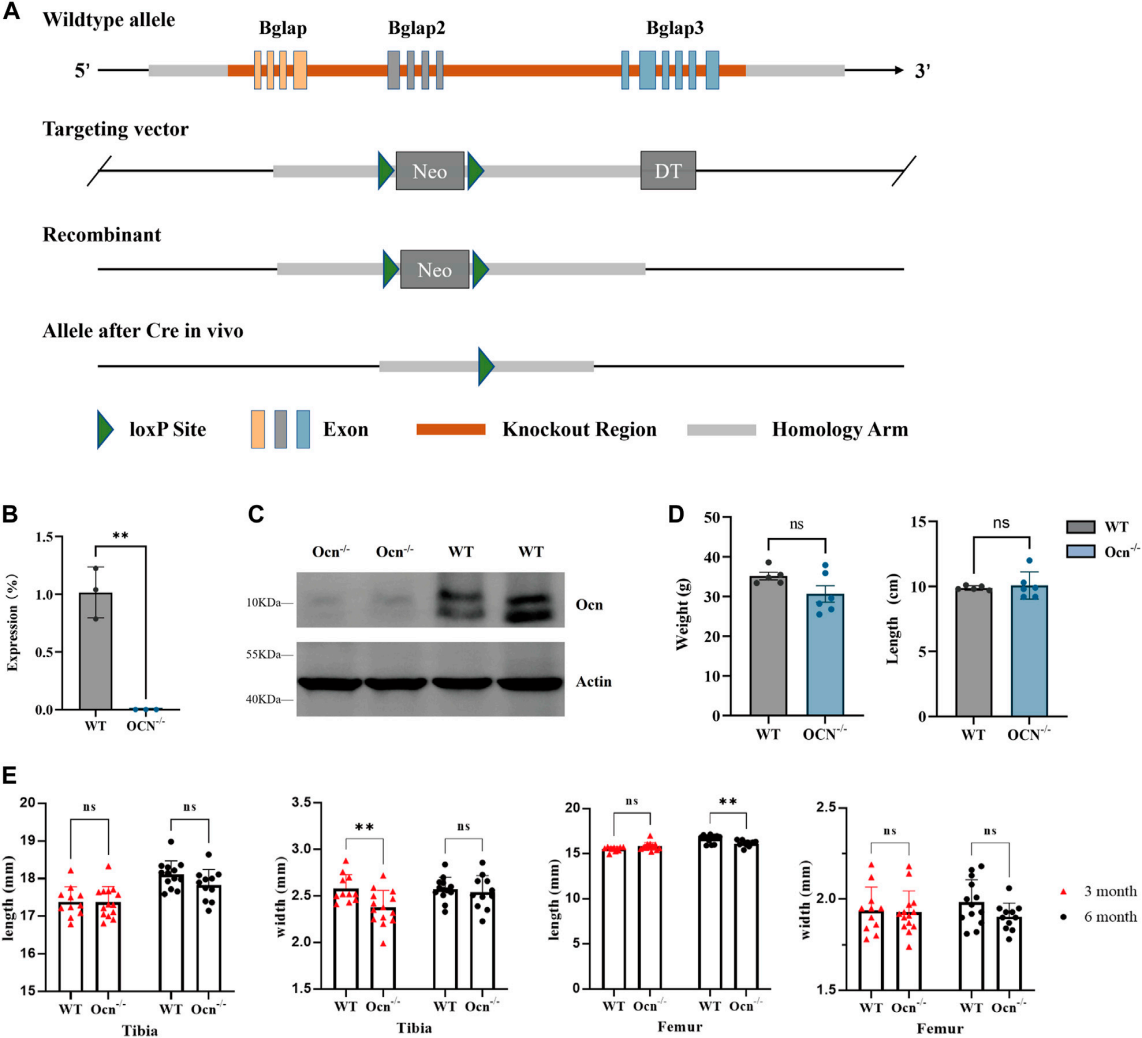
Generation of Ocn^−/−^ mice and bone phenotype analysis. **(A)** Schematic presentation of Bglap, Bglap2, and Bglap3 gene loci, targeting vectors, and knockout region. **(B)** mRNA expression of Ocn in the femur was determined using quantitative RT-PCR (**: *p* < 0.01, n = 3). **(C)** Protein expression of Ocn in the femur was measured by Western blot analysis. **(D)** Body weight, and body size for 6-month-old WT (n = 5) and Ocn^−/−^ male mice (n = 6). **(E)** Length and width of tibia and femur phenotype parameters in 3-month-old and 6-month-old Ocn^−/−^ male mice compared with WT male mice (**: *p* < 0.01, ns: *p* > 0.05, n = 11).

Ocn KO mice, which possess a loxP site flanking Bglap, Bglap2, and Bglap3 genes, were generated using the homologous recombination method (Cyagen Biosciences, Inc., Sunnyvale, CA). The targeting strategy allows the generation of conventional knockout mice with Bglap- Bglap2- Bglap3 genes. Briefly, a homology region covering mouse Bglap to Bglap3 was subcloned into the targeting vector. One Loxp site was introduced into the Bglap promotor region, and another Loxp site together with a modified Rox-flanked Neo cassette was introduced into the region after Bglap3 (Figure 1A). The 5′homology arm and 3′homology arm were amplified from BAC (RP24-171H19) DNA and confirmed by end sequencing. After linearization, the targeting vector was transfected into C57BL/6N background mouse embryonic stem cells. The positive clones were selected by G418 and injected into mouse blastocysts, which were then implanted into pseudo-pregnant females. F0 chimeric mice were further bred to C57BL/6N wild-type mice to generate F1 heterozygous mice. Ocn^+/−^ mice were generated by crossing Ocn^flox/flox^ mice with CMV-Cre mice (B6.C-Tg (CMV-cre)1Cgn/J). Male and female heterozygous mice were crossed to generate homozygote Ocn^−/−^ mice, and Wild-type control mice (WT) were derived from littermates. The expression of Ocn was analyzed by RT-PCR. All test items used male mice for experimentation. Before the study, all experiments were reviewed and approved by the Animal Care and Use Committee of China Astronaut Research and Training Center (No. ACC-IACUC-2021-032). Animals were housed in a specific-pathogen-free environment on a 12-h light cycle at 22°C ± 2°C temperature-controlled conditions. All the animals were supplied with food and water and were sacrificed by cervical dislocation at respective time points.

### 2.2 Real-time RT-PCR analysis and Western blot analysis

The total femur bone tissues were ground under liquid nitrogen and RNA was extracted by TRIzol (Invitrogen, Waltham, MA, United States), and then reverse transcription into cDNA was performed using a PrimeScript RT reagent kit (Takara, Dalian, China). Quantitative real-time PCR was performed using TB Green Premix Ex Taq II (Tli RNaseH Plus). The primer sequences of Ocn were designed to encompassing both of the three genes. Primers were designed using Primer 3. Ocn: Forward 5′- TGA​CAA​AGC​CTT​CAT​GTC​CA -3′, Reverse 5′- TAG​TGA​TAC​CAT​AGA​TGC​GT -3’; Actin: Forward 5′- TCA​GAT​GAA​TTT​TCG​TTG​GCA​GA -3′, Reverse 5′- GAG​CAC​TTG​GGT​TAC​TCC​ACG -3’. We used WT and KO primers to identify wild-type and Ocn knockout mice, respectively. Mice with both primer bands were heterozygous. WT: Forward 5′- ATG​AGG​GAG​ACA​ACA​GGG​AGG​AA -3′, Reverse 5′- TTT​GCT​GAT​TTG​TGT​GTT​TGG​ATG​T -3’; KO: Forward 5′- AGC​ATC​CTT​TGG​GTT​TGA​C -3′, Reverse 5′- AGG​GAG​GCA​TTC​CTG​TTG​G -3’.

For Western blot analysis, The total femur bone tissues were ground under liquid nitrogen and then lysed by RIPA buffer containing protease inhibitor cocktail (Roche, German). The membrane was blocked with 5% BSA in Tris-buffered saline containing 0.1% Tween 20 (TBST) for 1 h and then incubated overnight. For the detection of osteocalcin, we used anti-osteocalcin Ab (Affinity Biosciences, Cincinnati, OH, United States) and anti-Actin Ab (Servicebio, Wuhan, China). Signals were detected using enhanced luminescence (Bio-Rad, United States).

### 2.3 Raman spectroscopy analysis (RS)

Femurs were harvested from 6-month-old mice (Ocn^−/−^ and WT mice). Specimens were processed for cold polymethyl methacrylate (PMMA) embedding. Subsequently, the femur was sliced transversely into 10 μm cross sections at position 7 (Figure 4A), and the rest of the femur was sliced into 10 μm longitudinal sections. Selected points evenly distributed throughout the sectioned tissue from each bone cross-section were analyzed by Raman spectroscopy (Renishaw in *via*, Wotton-under-Edge, Gloucestershire, United Kingdom). The test points on the longitudinal section were at position 7, and each of the upper and lower cortical bone sections has two points side by side. A 785 nm laser was used to excite the electrons within the material, and 50× objective and the numerical aperture of 0.75 produced a laser spot of approximately 2 μm in diameter. The acquisitions were made in the spectral range of 100 cm^−1^–3500 cm^−1^, with an integration time of 30 s and one accumulation. Three spectra were acquired for each point, and 4 points were measured for every 24 samples. Normalization and baseline subtraction was performed on all ranges to obtain a pure bone tissue Raman spectrum signal. Raman spectrum showing the assignments for ν_1_PO_4_ (961–962 cm^−1^), ν_2_PO_4_ (430–431 cm^−1^), Amide I (1667–1670 cm^−1^), Amide III (1248–1252 cm^−1^), Proline (855–858 cm^−1^).

### 2.4 Scanning electron microscopy analysis (SEM)

Scanning electron microscopy analysis of mice femur sections was performed on a Hitachi S4800 high-resolution field-emission scanning electron microscopy with an accelerating voltage of 3.0 kV. Secondary electrons were obtained using a through-lens detector and were recorded at a 7.6 mm and 8.0 mm working distance. ImageJ software was used to analyze the gap area proportion of the transverse cross-section at position 7.

### 2.5 Fourier transform infrared spectroscopy (FTIR)

Femurs were harvested from 6-month-old mice (Ocn^−/−^ and WT mice) and then embedded with cold polymethyl methacrylate (PMMA). The samples were sliced transversely into 10 μm cross sections at position 7 and position 9 (Figure 4A). For FTIR analysis, Six selected points were randomly distributed throughout the sectioned tissue analysis (Nicolet iN10, Thermo Scientific, Waltham, MA, United States). Prior to analysis, the scanning range was set to 450–7,600 cm^−1^ with a spectral resolution of 4 cm^−1^. Signals were acquired every 2 min, and the field of view was 100 μm^2^ (10 μm*0 μm). The indexes were calculated by the following: mineralization = mineral (916–1180)/matrix (1588–1712); carbonate/matrix = carbonate (840–890)/matrix (1588–1712); carbonate/mineral = carbonate (840–890)/mineral (916–1180); collagen maturity = 1667/1685; crystallinity = 1030/1020; carbonate substitution = TypeA (878–879), TypeB (871–893), TypeL (865–867); HPO_4_/mineral = 1106/961, 1075/961, 1145/mineral (Courtland et al., 2008).

### 2.6 Micro CT (μCT)

The skin was removed from the mouse femurs and they were fixed in 70% ethanol. The whole secondary spongiosa of the left distal femur from each mouse was scanned *ex vivo* using a micro CT system (mCT40, SCANCO MEDICAL, Switzerland). The micro CT analysis followed the ASBMR standards (Bouxsein et al., 2010). The micro CT scan acquisition parameters were: voxel size = 39 μm3, X-ray energy = 70 keV, X-ray tube = 70 kVp, X-ray intensity = 114 μA, integration time = 200 ms. A total of 850 slices with a voxel size of 10.5 μm were scanned in the distal femur. Eighty slices of cortical and trabecular bone at position 9 (proximal to the distal growth plate) and eighty slices of cortical bone at position 7 (2.5 mm from the growth plate) were selected for analysis. The parameters cortical thickness (Ct.Th), bone volume fraction (BV/TV), trabecular number (Tb.N), trabecular thickness (Tb.Th) and trabecular separation (Tb.Sp) were calculated from the three-dimensional reconstruction of the selected slices.

### 2.7 Tail suspension experiment

The Tail suspension manipulation was performed as Wronski described previously (Wronski and Morey-Holton, 1987) with minor modifications as we used before (Zhang et al., 2020). Six-month-old male mice were suspended by the tail attached to a chain hanging from a beam using a strip of adhesive surgical tape and maintained one per cage while they were tail-suspended. The bodies of mice were at a 30° angle to the floor, with only the forelimbs touching the floor and allowed to freely move to food and water. The mice were sacrificed by rapid cervical dislocation after hindlimb unloading for 28 days. FTIR and μCT were used to analyze bone minerals and microstructure before and after tail suspension in Different cohorts of mice.

### 2.8 Data analyze

Indexes statistical analyses were performed using the Student's t-test to compare WT and Ocn^−/−^ or using two-way ANOVA to compare WT and Ocn^−/−^ and their tail-suspended test groups in μCT analysis by Prism 9.0 (GraphPad Prism software). Data were presented as mean ± standard deviation (SD). The Raman spectroscopy regression models were built using the Huber-loss function (Huber Regressor) with python v3.7 (Python Software Foundation). Statistical in RS was analyzed by comparing the Δy value (distance value y from each point to the fitted curve) between the two genotype groups using a *t*-test. For FTIR in Figure 5, the data was displayed in the form of the percentage change. Percentage (%) was calculated by dividing each value by the average value of the WT control group.

## 3 Results

### 3.1 Physiological measurements of femur phenotype in Ocn^−/−^ mice

To generate Bglap, Bglap2, and Bglap3 triple-knockout mice, genomic DNA encompassing all three genes was replaced with the neo gene (Figure 1A). Real-time RT-PCR analysis indicated that the expression of Ocn was absent in Ocn^−/−^ mice using an Ocn triple-gene consensus segment primer (Figure 1B; Supplementary Figure S1). The expression of proteins was assessed by Western blotting, and Ocn proteins were also absent in Ocn^−/−^ mice (Figure 1C). There were no significant differences in body weight and body length between WT and Ocn^−/−^ male mice (Figure 1D). We then measured the bone phenotype of the Tibia and Femur in 3-month and 6-month-old mice. The Tibia width significantly decreased in 3-month-old Ocn^−/−^ mice. However, there was no intergroup difference between 6-month-old Ocn^−/−^ and WT mice. The Femur length significantly decreased in 6-month-old Ocn^−/−^ mice. No significant differences were observed between the other comparison groups (Figure 1E).

### 3.2 Ocn deficiency results in the disordered alignment of collagen fibers and hydroxyapatite crystals in bone

**FIGURE 2:**
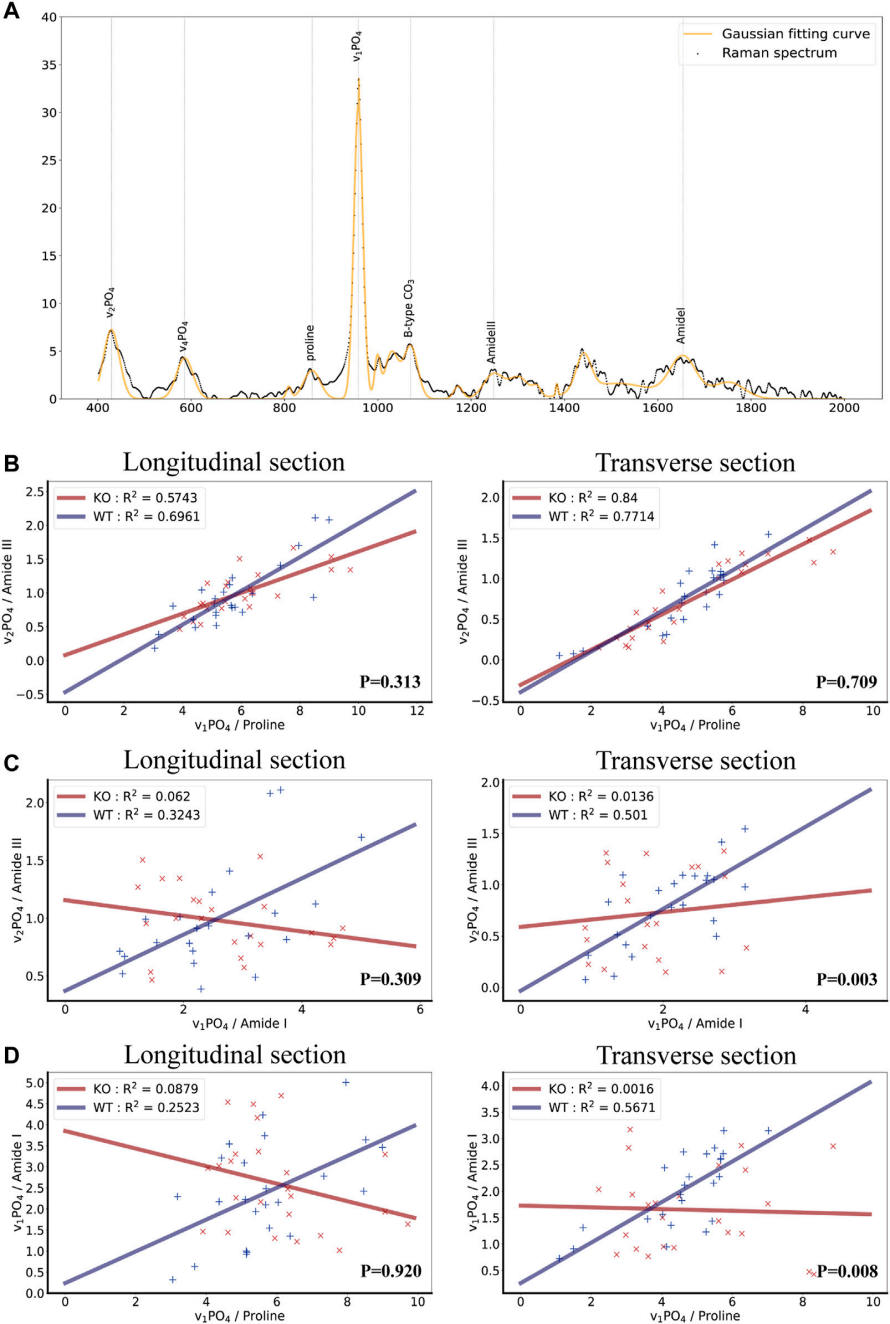
Biochemical properties of bone as measured by Raman spectroscopy in wild-type and Ocn^−/−^ mice. **(A)** Raman spectrum and Gaussian fitting curve of bone tissue showing the major bands and the corresponding compounds. The background signal has been removed. **(B)** Scatter plots, fitted lines, and correlation coefficient (*R* ^2^) of ν_1_PO_4_/Proline and ν_2_PO_4_/AmideIII groups in both WT and Ocn^−/−^ (KO) male mice (Left: longitudinal section, Right: Transverse section, n = 6). **(C)** Corresponding analysis of ν_2_PO_4_/AmideIII and ν_1_PO_4_/AmideI groups in both WT and Ocn^−/−^ (KO) male mice (Left: longitudinal section, Right: Transverse section, n = 6). **(D)** Corresponding analysis of ν_1_PO_4_/Proline and ν_1_PO_4_/AmideI groups in both WT and Ocn^−/−^ (KO) male mice (Left: longitudinal section, Right: Transverse section, n = 6). (P: comparing the Δy value (distance value y from each point to the fitted curve) between the two genotype groups using a *t*-test).

Raman spectroscopy (RS) can be used as a complementary tool for bone diagnosis due to its ability to assess compositional and organizational characteristics of both collagen and mineral. As described in the calculation method section, the ratio of organic and inorganic peaks was compared, and linear fitting was performed to judge the changes of collagen and hydroxyapatite arrangement angle in bone (Figure 2A). Three types of calculations for mineral/matrix ratios can be described as follows: mineral/matrix A=(ν_1_PO_4_/AmideI) γ (where γ is angle coefficient), mineral/matrix B = ν_2_PO_4_/AmideIII, mineral/matrix C = ν_1_PO_4_/Proline (Makowski et al., 2013). The ratio of ν_1_PO_4_/AmideI ratio varied upon changing the measurement angles of the RS analysis. When the angle between hydroxyapatite crystals and collagen is uncertain, γ cannot be calculated, and the accurate mineral matrix ratio through ν_1_PO_4_/AmideI can not be drawn. However, the ratio of ν_1_PO_4_/Proline or ν_2_PO_4_/AmideIII are angle-insensitive, and mineral/matrix B and mineral/matrix C can accurately describe the mineral matrix ratio. In that case, every test point of mineral/matrix B and mineral/matrix C had an excellent linear fitting, but mineral/matrix A could not. Therefore, the linear fit between each of the two ratios could be used to calculate whether the alignment between hydroxyapatite crystals and collagen is consistent.

We calculated the three ratios in the bone longitudinal section and transverse cross-section and performed a linear fit between any two ratios using the Huber loss function (Huber Regressor). The results revealed a high fit of ν_1_PO_4_/Proline and ν_2_PO_4_/AmideIII groups in both WT and Ocn^−/−^ male mice as expected (Figure 2B). Impressively, the *R* ^2^ (goodness-of-fit measure for linear regression models) of the linear regression model of ν_2_PO_4_/AmideIII and ν_1_PO_4_/AmideI was much lower in Ocn^−/−^ mice than in WT mice (Figure 2C). The *R* ^2^ of the linear regression model of the ν_1_PO_4_/Proline and ν_1_PO_4_/AmideI was also lower in Ocn^−/−^ mice (Figure 2D). These results suggested that the alignment of collagen fibers and hydroxyapatite crystals was disordered in Ocn^−/−^ mice.

### 3.3 SEM images analyzed showed a significant increase in gap area in the ocn^−/−^ mice

**FIGURE 3:**
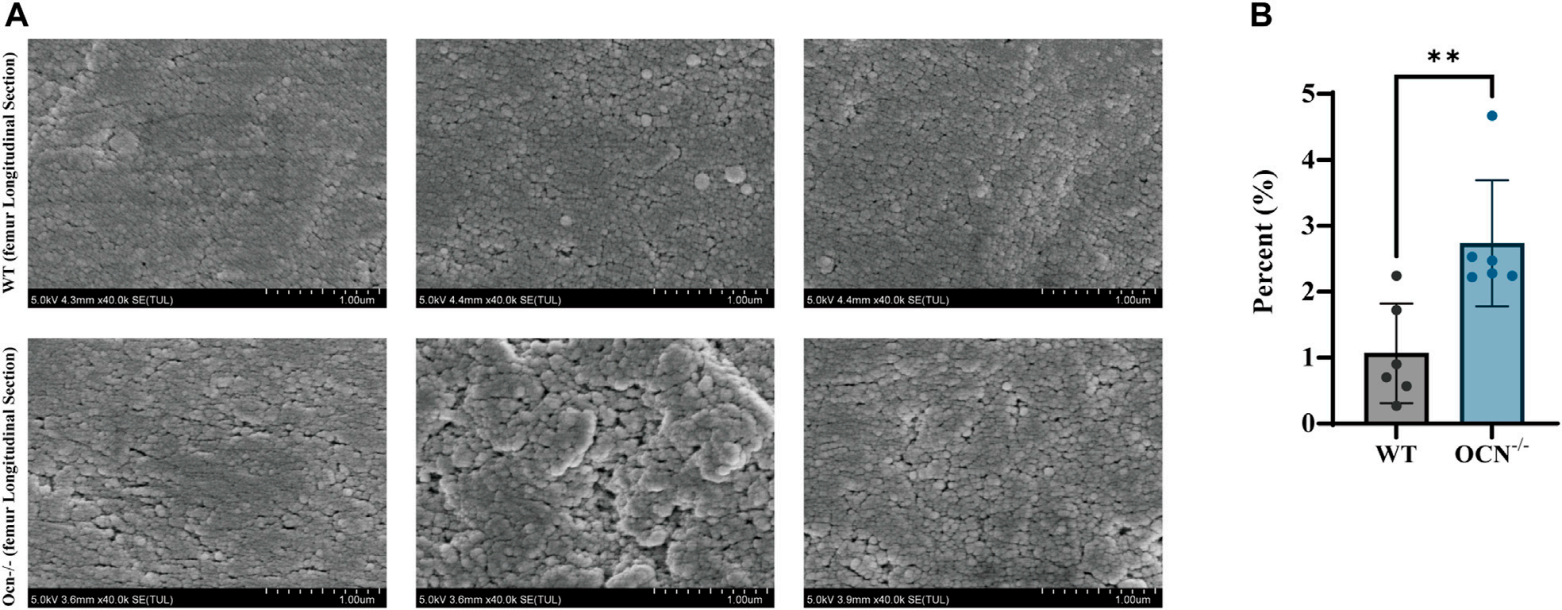
SEM images of the femur in the transverse cross-section between WT and Ocn^−/−^ mice. **(A)** SEM images of cortical bone show the variable’s spatial distribution in WT and Ocn^−/−^ male mice. **(B)** Histogram of gaps area proportion from femur section between male 6-month-old WT (gray column) and Ocn^−/−^ male mice (blue column) at position 7. (*: *p* < 0.05, ns: *p* > 0.05, n = 6).

A commonly used technique for imaging bone structure from microscale to sub-cellular scales is scanning electron microscopy (SEM). The standard SEM can provide high-resolution images on the order of a few nm (2–10 nm) (Ambekar et al., 2012). To investigate the effects of collagen arrangement on the bone structure, we used SEM to analyze the femur longitudinal section morphology in a position consistent with the RS scan. As shown in Figure 3A, a looser surface structure of bone particles was observed in the Ocn^−/−^ male mice compared with WT male mice. We also counted the area and perimeter of the gaps between particles (Supplementary Figure S2). There was a significant increase in gap area proportion in the Ocn^−/−^ mice, indicating a large and irregular gap between the femoral microstructures (Figure 3B).

### 3.4 Fourier transform infrared spectroscopy (FTIR) analysis showed few differences in bone mineral index between WT and ocn^−/−^ mice

**FIGURE 4:**
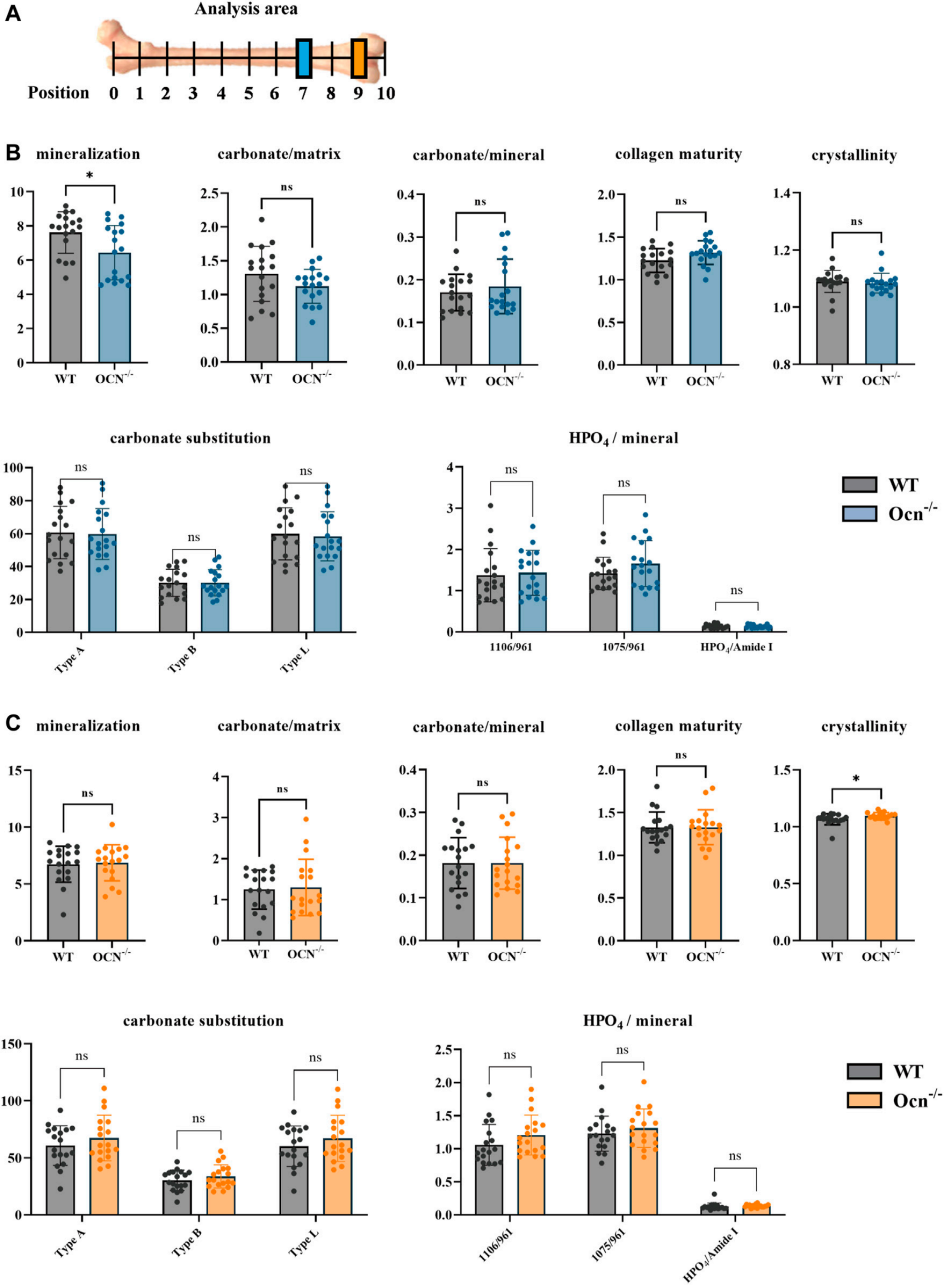
An FTIR analysis showed few differences in bone mineral index between WT and Ocn^−/−^ mice. **(A)** Schematic presentation of analyzed positions of bone section used for FTIR (Color annotation for Position 7 (WT: grey, Ocn^−/−^: blue) and Position 9 (WT: grey, Ocn^−/−^: orange)). **(B)** FTIR analysis of position 7 femur section at 6 months of age. The mineralization, carbonate/matrix, carbonate/mineral, collagen maturity, crystallinity, carbonate substitution (3 algorithms: typeA, typeB, typeL), and HPO_4_/mineral were compared between WT and Ocn^−/−^ male mice. **(C)** FTIR analysis of position 9 femur section at 6 months of age. The analysis indexes were the same as the detection indexes in position 9 compared between WT mice and Ocn^−/−^ male mice. (*: *p* < 0.05, ns: *p* > 0.05, n = 18).

The chemical aspects of the bone matrix were analyzed in the posterior part of the femurs (Position 7 and Position 9) by FTIR (Figure 4A). Seven indexes including mineralization, carbonate/matrix, carbonate/mineral, collagen maturity, crystallinity, carbonate substitution (3 algorithms: typeA, typeB, typeL), and HPO_4_/mineral were used for assessing the constitution of the bone matrix between WT and Ocn^−/−^ male mice in bone sections (Faibish et al., 2005; Courtland et al., 2008). There was a significant decrease (*p* < 0.05) in mineral/matrix at position 7 and a significant increase (*p* < 0.05) in crystallinity at position 9 in the Ocn^−/−^ mice compared with WT mice. No significant differences were found in other indexes between the two groups (Figures 4B, C).

### 3.5 The bone mineral content changed more variable in ocn^−/−^ mice than in WT mice under the tail suspension test

**FIGURE 5:**
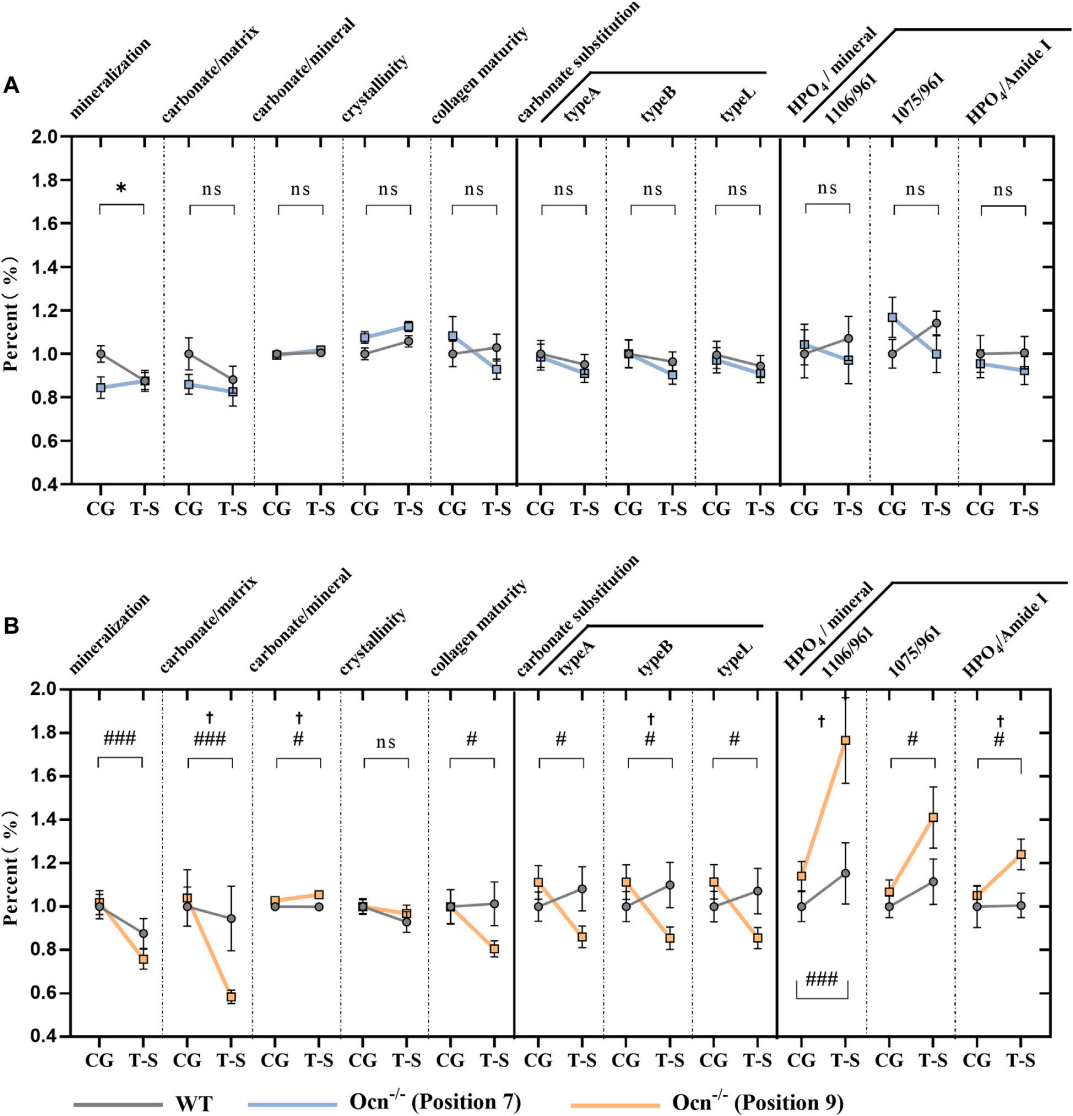
FTIR analysis of femur bone between WT and Ocn^−/−^ mice pre- and post-tail suspension test. **(A)** FTIR analysis of femur section at position 7 pre- and post-tail suspension test. The mineralization, carbonate/matrix, carbonate/mineral, collagen maturity, crystallinity, carbonate substitution (3 algorithms: typeA, typeB, typeL), and HPO_4_ substitution were tested pre- and post-tail suspension test in WT (grey line) and Ocn^−/−^ male mice (blue line). **(B)** FTIR analysis of femur section at position 9 pre- and post-tail suspension test in WT (grey line) and Ocn^−/−^ male mice (orange line). (CG: control group, T–S: tail-suspension group,: *p* < 0.05 (CG and T-S group compared in WT male mice), #: *p* < 0.05 (CG and T-S group compared in Ocn^−/−^ male mice), ##: *p* < 0.01 (CG and T-S group compared in Ocn^−/−^ male mice), ###: *p* < 0.001 (CG and T-S group compared in Ocn^−/−^ male mice), †: *p* < 0.05 (WT and Ocn^−/−^ compared after tail suspension), ns: *p* > 0.05, n = 18).

The tail suspension was performed to achieve an unloading status of hindlimbs (Cryan et al., 2005). This animal model can be used to analyze the effect of Ocn deficiency on bone during unloading. After 28 days of the tail-suspension test, FTIR parameters of femur microarchitecture were measured using the previously described methods in WT and Ocn^−/−^ male mice. Except for mineralization value in WT mice, we found no significant difference in other FTIR indexes at position 7 during the experiment (Figure 5A). However, mineralization, carbonate/matrix, collagen maturity, and crystallinity significantly decreased (*p* < 0.05) in Ocn^−/−^ mice compared with WT mice. These results indicated that Ocn deficiency accelerates mineral loss. Regarding crystal replacement, carbonate substitution was significantly decreased at position 9 in the Ocn^−/−^ mice. Meanwhile, each index of HPO_4_/mineral significantly increased, and this change was less pronounced in WT mice (Figure 5B). Therefore, the inconsistent arrangement of hydroxyapatite and collagen may decrease hydroxyapatite stability, and Ocn knockout mice were more likely to have crystal replacement during bone loss.

### 3.6 Effect of Ocn knockout on the bone microstructure caused by the tail suspension

**FIGURE 6:**
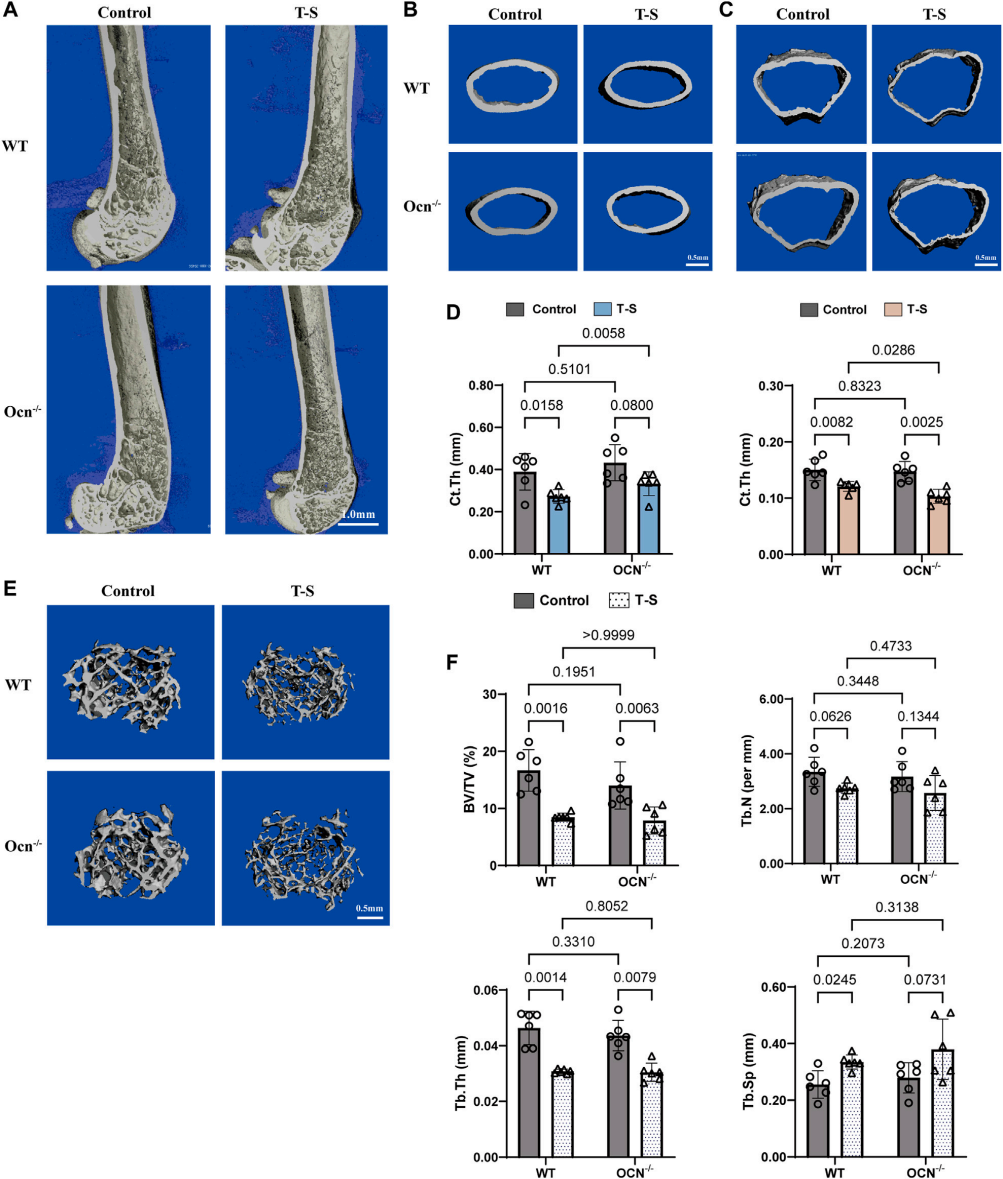
μCT analysis of femur bone before and after tail suspension test. **(A–C)** Representative 3D μ-CT images of distal femur and cortical regions from 6-month-old WT and Ocn^−/−^ male mice (Scale bars: 1 mm for the femur and 0.5 mm for cortical regions). **(D)** µCT analysis of Ct. Th in cortical regions at position 7 and position 9 from WT and Ocn^−/−^ mice before and after tail suspension test (Control: control group, T-S: tail-suspension group, n = 6). **(E)** Representative 3D μ-CT images of trabecular regions from 6-month-old WT and Ocn^−/−^ male mice (Scale bars: 0.5 mm). **(F)** µCT analysis of BV/TV, Tb.N, Tb. Sp and Tb.Th in trabecular regions at position 9 from WT and Ocn^−/−^ mice before and after tail suspension test (Control: control group, T-S: tail-suspension group, n = 6).

To examine the effect of Ocn knockout-mediated alteration of microstructural morphology in bone, we used μCT scanning to quantify the changes in the bone microstructure of the WT and Ocn^−/−^ male mice and their tail-suspended test groups (Figures 6A–C). We analyzed the thickness of cortical (Ct.Th) of cortical regions at position 7 and position 9, and BV/TV, trabecular (Tb.N), thickness (Tb.Th) and separation (Tb.Sp) of trabecular regions at position 9. The scanning results demonstrated that these indexes have no significant difference between WT and Ocn^−/−^ mice before the tail suspension (Figures 6D, F). After 28 days of tail suspension, the cortical bone thickness at position 7 and 9 decreased significantly (Figure 6D), and the reduction ratio was greater in Ocn^−/−^ mice than in WT mice at position 9 (Supplementary Figure S3A, B). The trabecular regions at position 9 showed significant bone loss after tail suspension, with BV/TV and Tb.Th decreased significantly, and Tb.N and Tb. Sp increased significantly (Figures 6E, F). However, there was no significant difference in the trabecular bone between the two groups of tail-suspended mice (Supplementary Figure S3C).

## 4 Discussion

In this study, we generated triple-gene knockout mice for Bglap, Bglap2, and Bglap3, which encode osteocalcin (Ocn). This is the first study to examine the effects of deleting all three Ocn genes in mice with a C57BL/6 substrain background. Our findings could complement previous studies using different Ocn knockout models. We measured bone mass and quality at two different sites of the femur using RS, SEM, FTIR, and mCT methods. We observed that the alignment of collagen fibers and hydroxyapatite crystals was disrupted in Ocn^−/−^ mice by RS analysis. The gap area proportion was significantly increased in Ocn^−/−^ mice, indicating large and irregular spaces between the femoral microstructures. The FTIR analysis showed that bone mineral index was similar between Ocn^−/−^ mice and wild-type (WT) mice. However, Ocn deficiency caused the misalignment of collagen fibers and hydroxyapatite crystals in bone. Mineral loss was more rapid in Ocn^−/−^ mice than in WT mice during tail suspension. The mCT scanning revealed that bone microstructure was comparable between WT and Ocn^−/−^ mice before tail suspension. After 28 days of tail suspension, the cortical bone thickness decreased more in Ocn^−/−^ mice than in WT mice at position 9. Taken together, these results suggest that Ocn regulates the organization of collagen fibers and hydroxyapatite crystals in bone.

Investigators have tried a variety of knockout mouse models to illuminate the function of Ocn. The first Ocn-deficient mice (osc^m1^/osc^m1^) were generated by simultaneously deleting both Bglap and Bglap2 using embryonic stem cell technology (Ducy et al., 1996). The Bglap3 gene was not deleted in this mouse model because the Bglap3 is expressed in the kidney but not in bone (Rho et al., 1998). With this model, the researchers investigated the function of Ocn in bone and then demonstrated the endocrine regulation role of Ocn in regulating whole-body physiology, such as energy metabolism, male fertility, and brain development (Lee et al., 2007; Oury et al., 2011; Oury et al., 2013; Mera et al., 2016; Zoch et al., 2016; Khrimian et al., 2017; Yadav et al., 2022). Due to the essential functions of Ocn, another Ocn^−/−^ mouse model was constructed with C57BL/6N genetic background. The only difference between the two mouse lines is that the previous Ocn^−/−^ mouse model was generated in the mixed background of C57BL/6J and 129/SV (Moriishi et al., 2020). Meanwhile, Diegel et al. generated Bglap/2^dko/dko^ by simultaneously injecting Cas9 protein and guide RNAs that recognize sequences within Bglap and Bglap2 but not within Bglap3 (Diegel et al., 2020). Although researchers broadly suggest that Bglap3 may have low expression in bone, it remains to be determined if there are any compensatory mechanisms when Bglap and Bglap2 gene is knockout. The functional significance of the Bglap3 allele in mice is also unclear. Therefore, we constructed an Ocn triple-gene knockout mice to complement the Ocn function study.

Ocn promotes normal bone mineralization by regulating the growth of hydroxyapatite crystals (Hooshmand and Arjmandi, 2009). Tsao et al. (2017) investigate the roles of Ocn in mineral species production during the osteogenesis of MSCs. Using Raman spectroscopy, they found that the maturation of mineral species was affected by the Ocn expression level. The mineral species maturation was delayed, and total hydroxyapatite was lower in the Ocn knockdown group than in the control group. The Ocn-derived peptide, particularly in amidic form, can act as a bioactive inducer of the mineralization process, hence accelerating bone tissue regeneration (Hosseini et al., 2019). These studies indicate that Ocn may play a role in maintaining bone mass. However, the absence of Ocn leads to an increase in bone formation without impairing bone resorption (Ducy et al., 1996). Using an Ocn^−/−^ rat model constructed by CRISPR/Cas9 technology, Lambert et al. (2016) found that complete loss of Ocn in the rat affected bone structure and function, with increased trabecular bone and increased bone strength. Thus, these contradictory results cast doubt on the role of Ocn as one of the main factors in bone mass. μCT and 3-point bending tests do not find differences in Bglap/2^dko/dko^ mice from wild-type littermates for bone mass and strength (Diegel et al., 2020), and Ocn is not involved in the regulation of bone quantity in Ocn-deficient mice (Moriishi et al., 2020). Our study investigates the function of Ocn in bone mass by μCT and found that bone mass was unchanged in the trabecular region after osteocalcin knockout. Until now, the study results remain conflicting, and further studies are warranted to confirm and explain the results.

The bone mineral is amorphous calcium phosphate (65% in young bone and 35% in adult bone), which is a precursor to crystalline hydroxyapatite (HAp), and fibrillar type I collagen provides a stable template for mineralization. Cross-linking is crucial for the stability and mineralization of collagen (Hakimimehr et al., 2005; Prabhakaran et al., 2009; Terajima et al., 2014). Thus, the proper amount and structure of the two matters play an essential role in bone formation and strength. Using small angle x-ray scattering (SAXS) and wavelength dispersive spectroscopy (WDS) on OC^−/-^ (Ocn^−/−^) genetic knockout mice bones, Poundarik et al. (2018) demonstrated that Ocn has specific roles in the biomolecular regulation of minerals in bone. Ocn is localized to both intrafibrillar and interfibrillar collagen. In the former, Ocn binds to type I collagen and may mediate nucleation, growth, and development of platelet-shaped apatite crystals within structural units of collagen such as microfibrils, fibrils and fibers (Chen et al., 2015). Another study suggested that Ocn was not necessary for the regulation of bone formation or resorption but essential for the alignment of the BAp c-axis parallel to collagen fibrils and required for optimal bone strength (Moriishi et al., 2020). These studies indicated that Ocn plays an essential role in mineral and collagen fibril arrangement. By using FTIR and RS methods, we found that the alignment of collagen fibers and hydroxyapatite crystals was more disordered in Ocn^−/−^ mice than in WT mice. Because of these structural features of binding calcium ions and hydroxyapatite, Ocn may have played a role in stabilizing the alignment of minerals and collagen in bone.

The tail-suspension mouse model is universally used to study unloading osteoporosis (Li et al., 2021; Camy et al., 2022; Zlotnick et al., 2022). We use the model to investigate the absence of Ocn on bone mass and bone minerals in the condition of bone loss. In the tail-suspended condition, Ocn knockout mice had a faster rate of mineral loss, and the proportion of carbonate substitution in the bone was also significantly reduced. Taken together, the loss of Ocn causes a disordered arrangement (low congruence) of collagen fibers and hydroxyapatite crystals, reducing the ability of the bone tissue itself to resist unloaded bone loss. In addition, we found that the No. 9 position of the femur of Ocn^−/−^ mice was more prone to acid phosphate substitution after tail suspension. It is important to note that male mice were mainly used in this study. Female Ocn knockout mice on a B6 background had a lower bone mineral density, and lower mineral-to-matrix ratio resulting in reduced stiffness and weaker bone strength. Suggest that reduced Ocn may contribute to fracture and weaker bone in some groups of elderly and adults (Berezovska et al., 2019). Moriishi et al. (2020) and Diegel et al. (2020) used both male and female Ocn knockout mice and found that the alignment of apatite crystallites, but not endocrine abnormalities, or muscle mass was changed in Ocn knockout mice (Manolagas, 2020; Moriishi and Komori, 2020). Taking into account the possible impact of gender, relevant research will be carried out in the future to explore the significance of this experimental result.

In conclusion, the alignment of hydroxyapatite crystals and collagen fibers showed inconsistency in Ocn^−/−^ mice. The disordered arrangement of hydroxyapatite will lead to changes in bone mineral composition in the case of tail suspension simulating bone loss.

## Data Availability

The raw data supporting the conclusion of this article will be made available by the authors, without undue reservation.
