# Correction: Triple-gene deletion for osteocalcin significantly impairs the alignment of hydroxyapatite crystals and collagen in mice

**DOI:** 10.3389/fphys.2026.1816216

**Published:** 2026-03-23

**Authors:** Zihan Xu, Chao Yang, Feng Wu, Xiaowen Tan, Yaxiu Guo, Hongyu Zhang, Hailong Wang, Xiukun Sui, Zi Xu, Minbo Zhao, Siyu Jiang, Zhongquan Dai, Yinghui Li

**Affiliations:** 1 School of Life Science and Technology, Harbin Institute of Technology, Harbin, China; 2 State Key Laboratory of Space Medicine Fundamentals and Application, China Astronaut Research and Training Center, Beijing, China; 3 Department of Pathology and Forensics, Dalian Medical University, Dalian, China

**Keywords:** osteocalcin, collagen fiber arrangement, bone mineral composition, bone microstructure, tail suspension

“C57BL/6 substrain background” was erroneously given as “C57BL/6J genetic background”.

A correction has been made to the section **4 Discussion**, Paragraph 1:

“In this study, we generated triple-gene knockout mice for Bglap, Bglap2, and Bglap3, which encode osteocalcin (Ocn). This is the first study to examine the effects of deleting all three Ocn genes in mice with a C57BL/6 substrain background. Our findings could complement previous studies using different Ocn knockout models. We measured bone mass and quality at two different sites of the femur using RS, SEM, FTIR, and μCT methods. We observed that the alignment of collagen fibers and hydroxyapatite crystals was disrupted in Ocn^−/−^ mice by RS analysis. The gap area proportion was significantly increased in Ocn^−/−^ mice, indicating large and irregular spaces between the femoral microstructures. The FTIR analysis showed that bone mineral index was similar between Ocn^−/−^ mice and wild-type (WT) mice. However, Ocn deficiency caused the misalignment of collagen fibers and hydroxyapatite crystals in bone. Mineral loss was more rapid in Ocn^−/−^ mice than in WT mice during tail suspension. The μCT scanning revealed that bone microstructure was comparable between WT and Ocn^−/−^ mice before tail suspension. After 28 days of tail suspension, the cortical bone thickness decreased more in Ocn^−/−^ mice than in WT mice at position 9. Taken together, these results suggest that Ocn regulates the organization of collagen fibers and hydroxyapatite crystals in bone.”

The original version of this article has been updated.

