# Supplementary material for: Triple-gene deletion for osteocalcin significantly impairs the alignment of hydroxyapatite crystals and collagen in mice
Source: Front Physiol. 2023 Mar 28;14:1136561. doi: 10.3389/fphys.2023.1136561 (PMC10089303; doi:10.3389/fphys.2023.1136561)
Supplement: Supplementary file 1 [file DataSheet1.PDF]

Supplementary Material

Triple-gene deletion for osteocalcin in mice significantly impairs the alignment of hydroxyapatite crystals and collagen

Zihan Xu[1,2†], Chao Yang[2†*], Feng Wu[2†], Xiaowen Tan[3], Yaxiu Guo[2], Hongyu Zhang[2], Hailong Wang[2], Xiukun Sui[2], Zi Xu[2], Minbo Zhao[3], Siyu Jiang[1], Zhongquan Dai[2*], Yinghui Li[1,2*]

*** Correspondence:** Zhongquan Dai, Yinghui Li, Chao Yang
daizhq77@163.com, yinghuidd@vip.sina.com, zhangxiuyc@sina.com.

1 Supplementary Figures

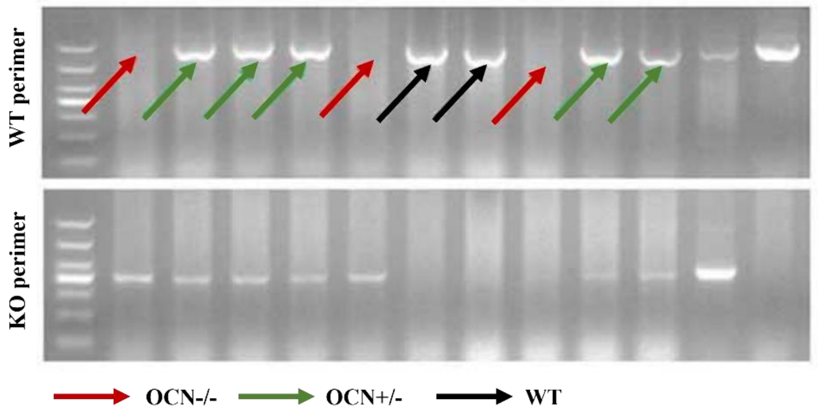

Supplementary Figure 1. Identification of mouse genotypes by PCR with the indicated primers. Red arrow: Ocn$^{-/-}$ mice, Green arrow: Ocn$^{+/-}$ mice, Black arrow: WT mice.

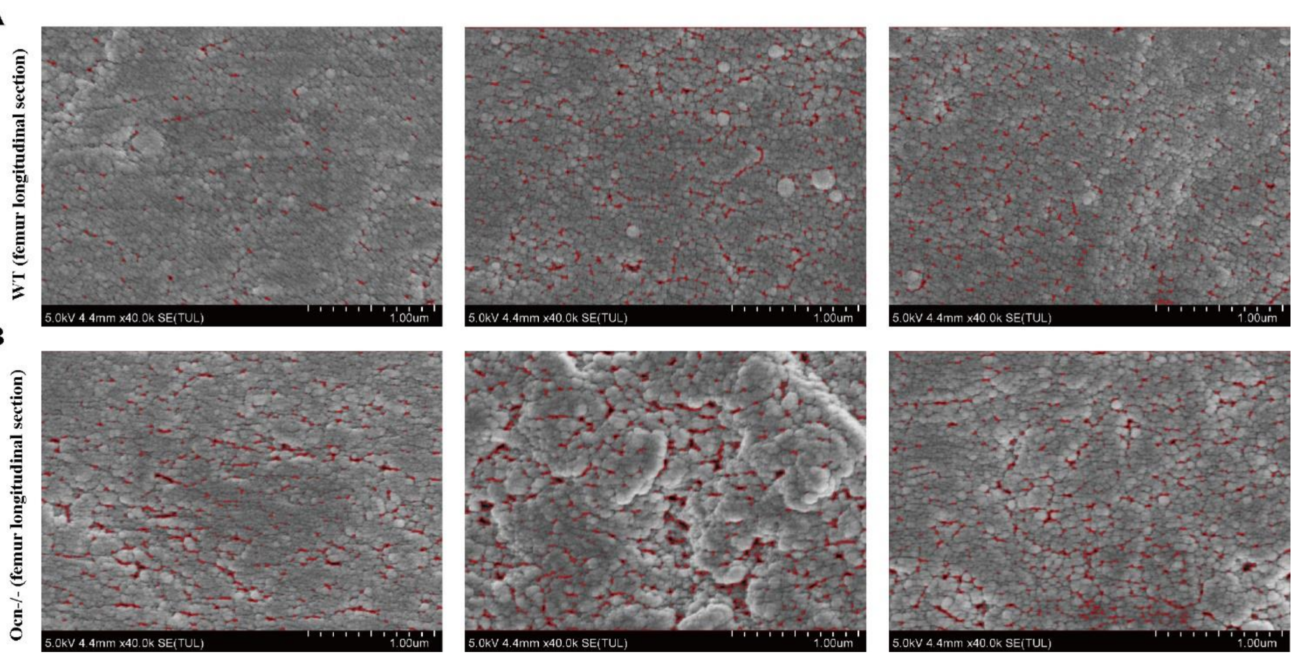

Supplementary Figure 2. Gaps area and perimeter statistics of SEM images. (A) Gaps area and perimeter analysis of SEM images in WT male mice. (B) Gaps area and perimeter analysis of SEM images in Ocn$^{-/-}$ male mice.

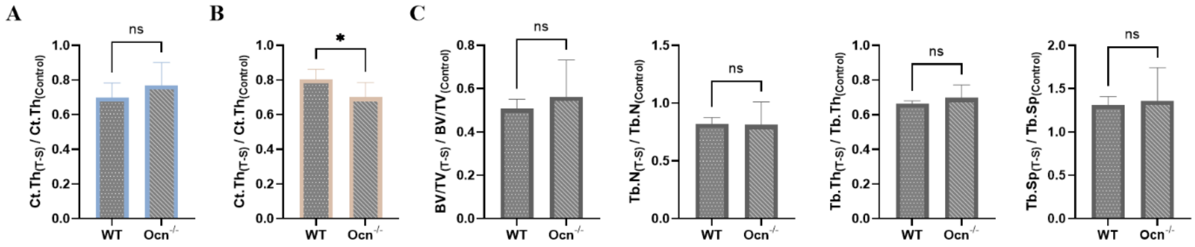

Supplementary Figure 3. Analysis of the change rate of microCT detection indexes before and after tail suspension experiment. (A) Change rate of Ct.Th in WT and Ocn$^{-/-}$ male mice in cortical regions at position 7. (B) Change rate of Ct.Th in WT and Ocn$^{-/-}$ male mice in cortical regions at position 9. (C) Change rate of BV/TV, Tb.N, Tb.Th, Tb.Sp in WT and Ocn$^{-/-}$ male mice in trabecular regions at position 9 (*: $P<0.05$, n=6).
